# De novo assembly of a new *Olea europaea* genome accession using nanopore sequencing

**DOI:** 10.1038/s41438-021-00498-y

**Published:** 2021-04-01

**Authors:** Guodong Rao, Jianguo Zhang, Xiaoxia Liu, Chunfu Lin, Huaigen Xin, Li Xue, Chenhe Wang

**Affiliations:** 1 State Key Laboratory of Tree Genetics and Breeding, Research Institute of Forestry, Chinese Academy of Forestry, Beijing, 100091 China; 2 Collaborative Innovation Center of Sustainable Forestry in Southern China, Nanjing Forestry University, Nanjing, 210037 China; 3 Key Laboratory of Tree Breeding and Cultivation, National Forestry and Grassland Administration, Research Institute of Forestry, Chinese Academy of Forestry, Beijing, 100091 China; 4 MIANNING Yuansheng Agricultural Science and Technology Co., Ltd., Liangshan Yi Autonomous Prefecture Mianning County, Sichuan, 615600 China; 5 Biomarker Technologies Corporation, Beijing, 101300 China

**Keywords:** Genomics, Genome

## Abstract

Olive (*Olea europaea* L.) is internationally renowned for its high-end product, extra virgin olive oil. An incomplete genome of *O. europaea* was previously obtained using shotgun sequencing in 2016. To further explore the genetic and breeding utilization of olive, an updated draft genome of olive was obtained using Oxford Nanopore third-generation sequencing and Hi-C technology. Seven different assembly strategies were used to assemble the final genome of 1.30 Gb, with contig and scaffold N50 sizes of 4.67 Mb and 42.60 Mb, respectively. This greatly increased the quality of the olive genome. We assembled 1.1 Gb of sequences of the total olive genome to 23 pseudochromosomes by Hi-C, and 53,518 protein-coding genes were predicted in the current assembly. Comparative genomics analyses, including gene family expansion and contraction, whole-genome replication, phylogenetic analysis, and positive selection, were performed. Based on the obtained high-quality olive genome, a total of nine gene families with 202 genes were identified in the oleuropein biosynthesis pathway, which is twice the number of genes identified from the previous data. This new accession of the olive genome is of sufficient quality for genome-wide studies on gene function in olive and has provided a foundation for the molecular breeding of olive species.

## Introduction

Olive (*Olea europaea* L.), belonging to the family Oleaceae, is one of the most important and widely distributed fruit trees in the Mediterranean Basin. It has a history of more than 4000 years and has been planted in more than 40 countries. China began importing olive seeds and seedlings from Albania in the 1960s and now cultivates olive trees in 14 provinces, mainly Gansu, Sichuan, and Yunnan. Olive oil is a world-famous high-grade cooking oil that is rich in unsaturated fatty acids and distinct micronutrients, such as oleuropein, squalene, and hydroxytyrosol^1^. Olive is also well known for its biological functions, including its anti-inflammatory, antiviral, cardiotonic, anti-carcinogenic, antioxidant, and antihypertensive properties^2,3^.

Olive has economic, ecological, cultural, and scientific value and is widely appreciated^4^. The selection of olive varieties has always been based on traditional breeding practices, thus rendering molecular breeding a challenge. This is an important contributor to the lack of availability of a high-quality genome. Thus far, the genomes of two olive varieties (*Olea europaea* L. subsp*. europaea* var*. europaea* cv. ‘Farga’ and *Olea europaea* L. *sylvestris*) have been sequenced^5,6^. The two versions of the genome are mainly based on the next-generation sequencing method, which generated genomes of 1.31 G and 1.48 G with contig N50 values of 52.35 kb and 25.49 kb, respectively. A large number of scaffolds were assembled from the contigs, but none of them were completely anchored to the chromosomes. It is relatively difficult to obtain high-quality plant genomes, as plant genomes are generally large, with high heterozygosity and high numbers of repetitive sequences^7^. Olive has high heterozygosity, high numbers of repetitive sequences, and a large genome, which has hindered the production of a high-quality reference assembly of the two versions of the olive genome. Technological improvements have increased the yield and length of genome sequencing, particularly third-generation sequence technologies, such as PacBio third-generation sequencing and Oxford Nanopore third-generation sequencing (ONT) technology^8,9^. In addition, a genome-wide chromosome conformation capture technique, Hi-C, is often used to further assemble chromosome-scale genomes based on a sequenced draft genome^10^.

**Fig. 1:**
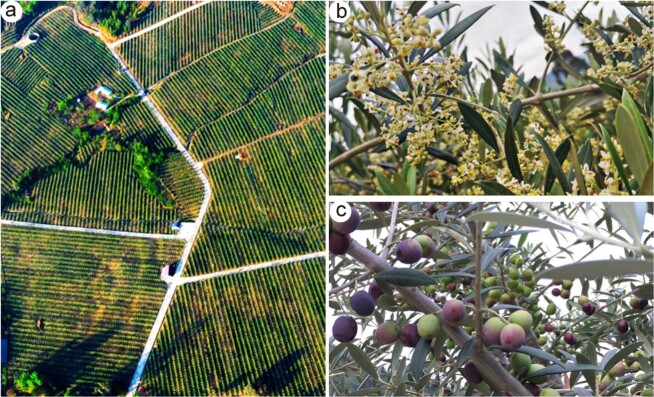
*Olea europaea* cv*. ‘Arbequina’*. **a** Intensive olive grove. **b** Flowering plant. **c** Olive fruits

In this study, an olive cultivar (*Olea europaea* L. subsp*. europaea* cv. ‘*Arbequina’*) that is suitable for mechanized harvesting and dense planting was sequenced using ONT sequencing (Fig. 1). Hi-C technology was used to generate a chromosome-scale assembly for the high-quality olive genome. We compared the results of different genome assembly strategies, namely, Canu, Wtdgb, and SMARTdenovo, with the single assembly; merged the assembled results in pairs; and merged the assembled results from the three methods. We discovered that SMARTdenovo had the best effect when using the single assembly strategy, while the strategy of merging the results of the three methods produced the longest contig N50 of 4.67 M. Using the obtained high-quality olive genome, we performed gene family expansion and contraction analysis, whole-genome replication analysis, phylogenetic analysis, positive selection analysis, and comparative genomics analysis.

## Results

### Preliminary characterization of the olive genome

**Fig. 2:**
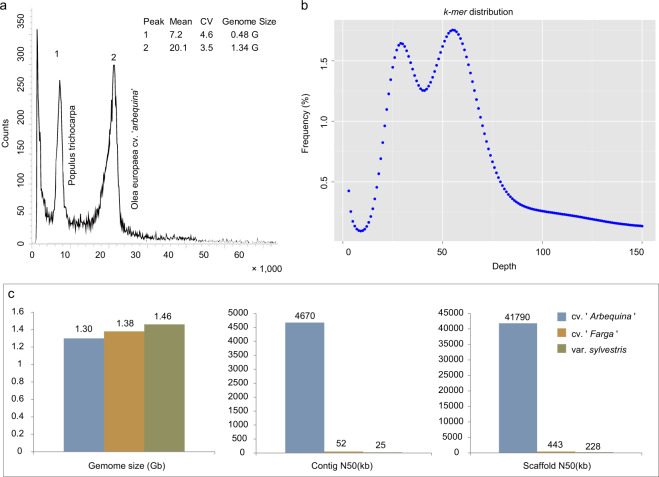
Preliminary characterization of the olive genome. **a** Flow cytometry. *Populus trichocarpa* was used as internal standard. Mean mean fluorescence per cell, CV coefficient of variation. The *x*-axis represents the relative content, and the *y*-axis corresponds to the number of events in graph **b**. *K-mer* analysis. The 21-mer frequency distribution derived from the sequencing reads was plotted. **c.** Comparison of three versions of the genome assembly

Due to the wide variety of olives, it was necessary to obtain information on the genome size, heterozygosity, and repeat content of this new accession of the olive genome. Three 350 bp libraries were constructed using genomic DNA from leaf samples, and 96.48 Gb of high-quality data was sequenced and filtered using the NovaSeq 6000 Illumina sequencing platform. The total sequencing depth was ~75×, and the sequencing data Q30 ratio was above 91.10% (Supplementary Table S1). Flow cytometry (Fig. 2b) and *k-mer* analysis (Fig. 2b) of this dataset indicated that the olive genome has a high level of heterozygosity (1.09%) with a repeat sequence content of 56.18% and a genome size of ~1.3G, which is slightly smaller than that of the previous olive genome (*Olea europaea* subsp. *europaea*; 1.38 GB)^6^ and oleaster genome (*Olea europaea* var. *sylvestris*; 1.46 GB)^5^.

### ONT sequence, genome assembly, and annotation

High-quality and high-molecular-weight genomic DNA was extracted and sequenced following ONT standard protocols^11^. A total of 9,009,932 raw reads with 146,825,799,392 bases were obtained. After further filtering out the adapters, low-quality reads, and short fragments (length < 2000 bp), the total dataset was obtained. Overall, we obtained 4,708,203 clean reads for a total of 129 Gb of sequence (representing 100×fold coverage; Supplementary Table S2). Notably, the average length of the reads was 27,311 bp, the length of read N50 values was 30,890 bp, and the longest read reached ~1 Mbp (962,647 bp). The clean read length distributions of all reads are shown in Supplementary Table S3. Most of the clean reads were distributed in the range of 20,000–50,000 bp, accounting for 77.08% of the total number of reads.

If DNA is contaminated, it will not only reduce the amount of valid data but also affect the accuracy of subsequent analyses and result in large deviations in genomic characteristics, such as the genome size, heterozygosity, repeat sequence ratio, and GC content, which will ultimately affect the subsequent genome assembly. If a certain proportion (1% or more) of reads match a species that is distantly related, the data may be contaminated. To determine whether the sequenced data were contaminated, we randomly selected 2000 reads from the sequencing data and performed a BLAST alignment with the nucleotide (Nt) database^12^, which showed that most of the reads were aligned to *O. europaea* (oleaster), *Hesperelaea palmeri*, *Vitis vinifera*, *Sesamum indicum*, and other plant species (Supplementary Table S4), verifying that the data were not contaminated. If the extranuclear DNA content in the sequencing library is too high, it will increase the difficulty of genome assembly and might even cause errors. We then performed a SOAP alignment with the three 350 bp libraries from the Illumina sequencing and the chloroplast sequence (NCBI Accession NO. NC_015623) of olive (Supplementary Table S5)^13^. Approximately 2–3% of reads were mapped to chloroplasts, and these sequences were removed before assembly. The results also showed that the enriched DNA was mainly olive nuclear DNA.

The sequenced ONT clean data were then assembled into the final genome using seven different assembly strategies, namely, Canu, Wtdgb, SMARTdenovo, Canu+Wtdgb, SMARTdenovo+Canu, Wtdgb+SMARTdenovo, and Wtdgb+SMARTdenovo+Canu, according to the standard protocols for each strategy (Table 1). Canu was used to precorrect the original reads^14^. A total of 1290 contigs were ultimately obtained by the combined Wtdgb+SMARTdenovo+Canu assembly strategy, with a contig N50 of 4.67 Mb and a total contig length of 1.30 G, and the largest contig in this assembly was 25.18 Mb. This is a great improvement over previous studies (with a contig N50 of 52.35 kb for *O. europaea* subsp. *europaea* and a contig N50 of 25.49 kb for oleaster)^5,6^ (Fig. 2c).

**Table 1:** Assembly statistics for the seven different assembly strategies

Method	Contig number	Contig length (kbp)	Contig N50 (bp)	Contig max (bp)	GC content (%)
Canu	4945	1,762,980	847,968	24,854,984	34.2
Wtdgb	7264	1,019,118	759,558	5,040,028	34.81
SMARTdenovo	2372	1,278,453	1,072,091	10,833,225	34.28
Canu + Wtdgb	3270	1,386,672	2,686,808	24,357,777	34.08
SMARTdenovo + Canu	2372	1,278,453	1,072,091	10,833,225	34.28
Wtdgb + SMARTdenovo	1459	1,164,886	3,113,447	11,036,205	34.34
Wtdgb + SMARTdenovo + Canu	1290	1,301,740	4,665,036	25,178,397	34.33

Three strategies were next used to evaluate the integrity of the assembled genome. First, 99.33% (656,154,460 of 660,556,220) of Illumina DNA-Seq reads were mapped to the assembled genome, and the properly mapped (paired-end reads mapped to the genome with a distance consistent with the length distribution of the sequenced fragments) read rate was 86.81%. BUSCO was first used to search the conserved plant genes (1614 conserved plant genes in the database) in the assembled olive genome, and 1521 genes, accounting for 94.24% of the total genes in the database, were identified (Supplementary Table S6). This ratio is similar to that for cv ‘*Farga’* (1501 genes account for 92.99% of the total genes in the database) but much higher than that for var. *sylvestris* (1380, accounting for 85.50% of the total genes in the database). BUSCO analysis of gene sets was also conducted in these three versions of the olive genome, which also showed a high ratio of 92.87% in cv ‘*Arbequina*’, much higher than that in var. *sylvestris* (85.25%)^5,6^ (Supplementary Table S6). Then, 438 conserved genes (95.63%) were identified in the 458 eukaryotic conserved sequences using CEGMA (Supplementary Table S7). These high mapping rates indicate the high integrity of the assembled olive genome^15^.

### Hi-C scaffolding

**Fig. 3:**
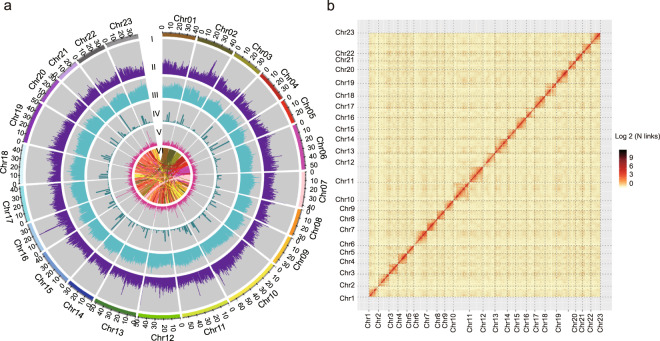
Chromosomal features and genome-wide all-by-all Hi-C interaction heatmap. **a** Chromosomal features. I, The 23 assembled chromosomes of the genome. II, Distribution of the GC content in the genome (purple). III, Repetitive sequences in the olive genome (cyan). IV, Distribution of gene density (bottle green). V, SSR (rose red). VI, Major interchromosomal relationships in the olive genome. All of these data are shown in 1 Mb with sliding windows of 500 kb. **b** Intensity signal heatmap of the Hi-C chromosome

A total of 232.97 Gb of clean data were obtained from Hi-C sequencing, covering the *O. europaea* genome at nearly 180x. After statistics and error correction of the genome sequences by Hi-C assembly, a total of 962 scaffolds, with a scaffold N50 of 42.60 Mb (Fig. 3a and Supplementary Table S8), were obtained. The derived scaffolds were then assembled into 23 chromosomes using LACHESIS analysis tools^16^. To assess the results of the Hi-C assembly, the 23 chromosomes were cut into equal lengths in 100 kb bins, and the number of Hi-C read pairs covered between any two bins was used as a signal of the strength of the interaction between the two bins. Twenty-three chromosomes could be clearly distinguished, and the intensity of the interaction at the diagonal position was higher than that at the nondiagonal position, indicating that the intensity of the interaction between adjacent sequences in the chromosome results of the Hi-C assembly was high, confirming that the assembled genome was of high quality (Fig. 3b). In total, 1.1 Gb of sequences was mapped onto the chromosomes. The sequences whose order and direction could be determined were 976.51 Mb, accounting for 95.03% of the total length of the mapped sequence (Supplementary Table S9).

Repeat sequences were predicted using the LTR_FINDER and RepeatScout software packages. A total of 1,815,585 sequences with a total length of 743,103,344 bp, accounting for 67.37% of the olive genome, were predicted (Fig. 3 and Supplementary Table S10). Genes were predicted using de novo, homologous species, and RNA-seq unigene prediction strategies. A total of 53,518 protein-coding genes were predicted on the current assembly, which has a similar number of gene sets to those in previous studies^5,6^. A genome-wide comparison was performed in the Rfam database. A total of 118 microRNAs and 192 rRNAs were identified using Blastn, and 674 tRNAs were identified using tRNAscan-SE^17^. Next, the predicted genes were annotated in the Gene Ontology (GO), Kyoto Encyclopedia of Genes and Genomes (KEGG), EuKaryotic Orthologous Groups (KOG), TrEMBL, and Nonredundant (Nr) databases. A total of 50,969 genes were annotated, accounting for 95.24% of the total predicted genes (Supplementary Table S11).

### Comparative genomics analysis

**Fig. 4:**
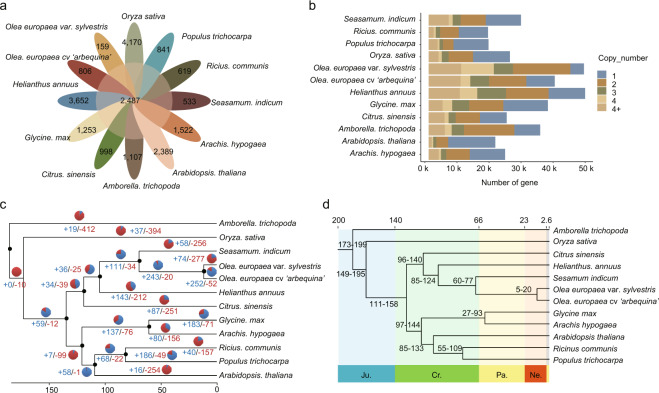
Gene families and phylogenetic analysis. **a** Petal diagram of the gene families of 12 species. The middle circle is the number of gene families shared by all species, and the number of gene families unique to each species is on the side. **b** Copy number distribution of the gene families of 12 species. **c** CAFE-based estimates of gene family expansions and contractions. The numbers after “+” and “−” represent the numbers of expanded and contracted gene families, respectively. The blue in the pie chart represents gene family expansion, and the red represents contraction. **d** Phylogenetic tree of *O. europaea* and 11 other species. At the bottom of the tree is geological time (prefix), and at the top of the tree is absolute age (measured in Mya). The tree is rooted with *A. trichopoda* as the outgroup. Ju. Jurassic, Cr. Cretaceous, Pa. Paleogene, Ne. Neogene

Comparative genomics analysis of *O. europaea* was performed with the genome sequences of 11 plant species (*Helianthus annuus*, *Glycine max*, *Arachis hypogaea*, *Ricinus communis*, *Arabidopsis thaliana*, *Populus trichocarpa*, *Sesamum indicum*, *Oryza sativa*, *Citrus sinensis*, *Amborella trichopoda*, and *Olea europaea* var. *sylvestris*). A total of 51,805 gene families were obtained; 2487 gene families were common among all 12 species, while 806 families were specific to olive (Fig. 4a and Supplementary Table S12). These specific gene families were then annotated to GO terms and KEGG pathways (Fig. S1). The GO annotations were mainly related to metabolic process, cellular process, and response to stimulus in the “biological process” term; cell part, cell, and organelle in the “cellular component” term; and catalytic activity, binding, and transporter activity in the “molecular function” term (Fig. S1a). The KEGG pathway analysis showed that “carbon metabolism” and “protein processing in endoplasmic reticulum” demonstrated the largest gene family expansion (Fig. S1b). Gene family copy number analysis showed that the olive gene family ranges from one to more than four copies, which is similar to that for sunflower and soybean, and olive has a large proportion of genes in families with four or more members (Fig. 4b). Further analysis of gene family expansion and contraction revealed that 252 gene families expanded and 52 gene families contracted in the olive genome (Fig. 4c). These 252 expanded gene families were then annotated to GO terms and KEGG pathways. The GO annotations were mainly related to response to stimulus, cellular process, and metabolic process in the “biological process” term; cell organelle, cell, and cell part in the “cellular component” term; and transporter activity, binding, and catalytic activity in the “molecular function” term (Fig. S2). The KEGG pathway analysis showed that “oxidative phosphorylation”, “photosynthesis”, and “plant-pathogen interaction” demonstrated the largest gene family expansion (Fig. S3).

**Fig. 5:**
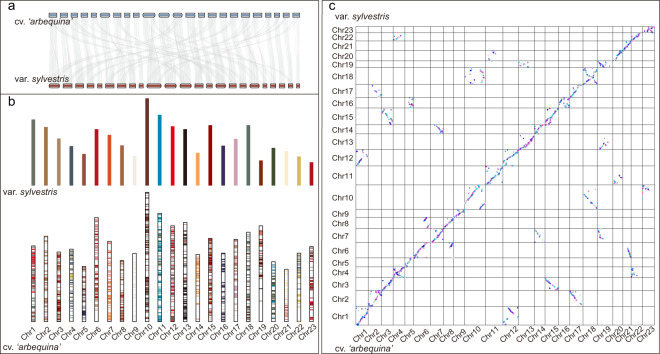
Synteny analysis between *O. europaea* cv*. ‘*A*rbequina’* and *O. europaea* var. *sylvestris*. **a** Linear synteny diagram of chromosomes. **b** Column synteny diagram of chromosomes. **c** Dot synteny diagram of chromosomes

Phylogenetic analysis was conducted using the single-copy protein sequences of 12 species. As expected, oleaster and olive had the closest genetic relationship and diverged from their ancestors at ~5–20 Mya. Synteny analysis was carried out on olive (*O. europaea*) and oleaster (*O. europaea* var. *sylvestris*), and the variations in genome structure and homologous gene pairs were analyzed. There was a high linear relationship between the olive and oleaster genes (Fig. 5a); a total of 52,991 genes were found to have synteny with oleaster in olive. The synteny between chromosomes was partially dislocated (Fig. 5b). This may be a result of only ~50% of oleaster sequences being anchored to the chromosomes.

**Fig. 6:**
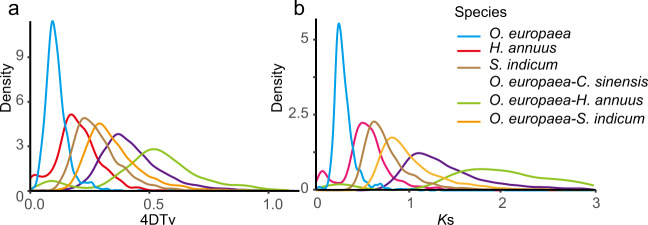
Whole-genome duplication (WGD) analysis. **a** 4DTv analysis. The x-coordinate is the 4DTv value, and the y-coordinate represents the proportion of genes corresponding to the 4DTv values. **b** *K*s distributions analysis. Peaks of intraspecies *K*s distributions indicate ancient whole-genome polyploidization events, and peaks of interspecies *K*s distributions indicate speciation events

Positive selection analysis identified 34 genes containing significantly positively selected sites. GO analysis showed that these genes were mainly in the “obsolete ATP catabolic process” category of the biological process term, “plasmodesma” in the cellular component category, and “organic cyclic compound binding” in the molecular function category. The KEGG pathway analysis revealed that these positively selected genes were mostly involved in pyruvate metabolism, nucleotide excision repair, and homologous recombination pathways. Whole-genome duplication (WGD) analysis was carried out by fourfold synonymous (degenerative) third-codon transversion (4DTv) and distributions of synonymous substitutions per synonymous site (*K*s). One main peak was observed in the *O. europaea* genome based on the abundance of 4DTv site values (4DTv value of 0.09) and *K*s value (*K*s value of 0.25), indicating that *O. europaea* had experienced a WGD event. The genomes of *C. sinensis*, *H. annuus*, and *S. indicum* were used to identify the 4DTv and *K*s values from synteny blocks between *O. europaea*, which suggested that *O. europaea* experienced large-scale gene duplication more recently than these three closely related species (Fig. 6).

### Identification of oleuropein and fatty acid biosynthesis genes in olive

**Fig. 7:**
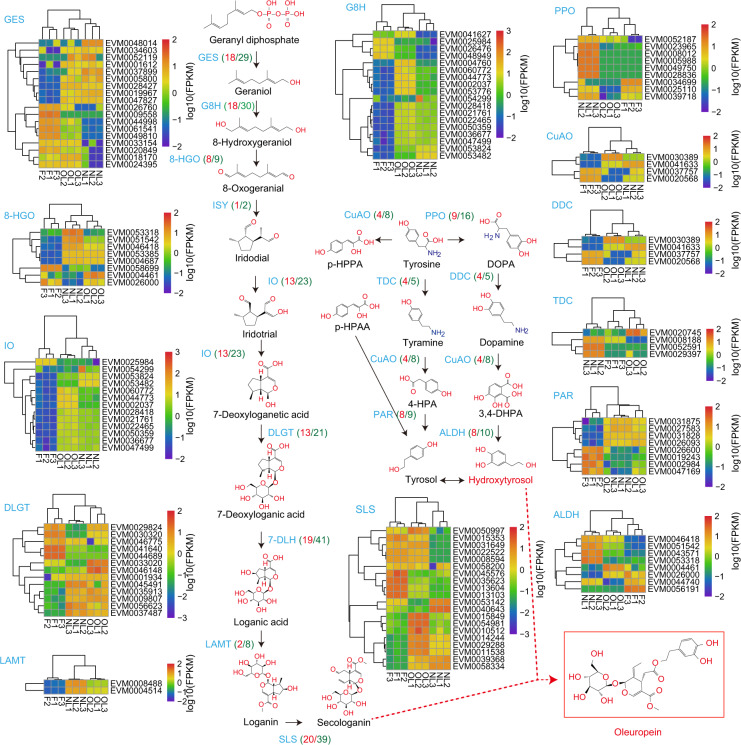
Identification and expression level of oleuropein and hydroxytyrosol biosynthesis genes in olive. Blue is the abbreviation for the gene, red numbers in brackets represent the number of detected genes in the gene family, and the green number in brackets is the number of total transcripts in the family. Heatmaps show the expression level of differentially expressed genes: yellow to red color represents higher than log_10_ (FPKM) data of genes; green to blue color represents lower than log_10_ (FPKM) data of genes. Samples are fruits (F1-F3 indicate the three biological replications), fully expanded leaves from shoots (NL1-NL3 indicate the three biological replications), and old leaves from the base of the stem (OL1-OL3 indicate the three biological replications). GES geraniol synthase, G8H geraniol 8-hydroxylase, 8-HGO 8-hydroxygeraniol oxidoreductase, ISY iridoid synthase, IO iridoid oxidase, 7-DLGT 7-deoxyloganetic acid-O-glucosyl transferase, 7-DLH 7-deoxyloganic acid hydroxylase, LAMT loganic acid methyltransferase, SLS secologanin synthase, PPO polyphenol oxidase, DDC DOPA decarboxylase, CuAO primary amine (copper-containing) oxidase, ALDH alcohol dehydrogenase, TDC tyrosine decarboxylase, PAR phenylacetaldehyde reductase, *p*-HPAA *p*-hydroxyphenylpyruvic acid, *p*-HPAA *p*-hydroxyphenylacetic acid, DOPA dihydroxyphenylalanine, 3,4-DHPA 3,4-dihydroxyphthalic acid, 4-HPA 4-hydroxyphenylacetic acid

Oleuropein and fatty acid biosynthesis pathway genes were identified based on their homology with known genes from transcriptome data^3^. A total of nine gene families with 202 genes in oleuropein biosynthesis and 14 gene families with 128 genes in the fatty acid biosynthesis pathway were identified, which is more than in the previous transcriptome data (Fig. 7). In terms of the oleuropein biosynthesis pathway, geranyl diphosphate is first catalytically converted to geraniol by 29 geraniol synthases (GESs), which is much more than the number identified in the previous study (four GES genes). Thirty geraniol 8-hydroxylase oxidoreductase (G8H) genes are involved in the hydroxylation of geraniol to 8-hydroxylase, which is twice the number of genes identified from the previous transcriptome data. 8-Hydroxyase is then catalyzed by 8-hydroxygeraniol oxidoreductase (8-HGO) to form 8-oxogeranial, and nine 8-HGO genes are involved in this step, which is less than the 13 genes previously identified. Iridoid synthase (ISY) forms iridodial from 8-oxogeranial, and two *ISY* genes were identified in this step, which is similar to the number identified in the earlier transcriptome study. Iridotrial and 7-deoxyloganic acids are synthesized as follows. The structural gene involved in this reaction is iridoid oxidase (IO), and 23 *IO* genes were identified in the olive genome, which is many more than in the transcriptome data. O-Glucosyl is then added to 7-deoxyloganic acid to form 7-deoxyloganic acid via the catalysis of 7-deoxyloganetic acid-O-glucosyl transferase (7-DLGT) and 21 7-DLGTs were identified. 7-Deoxyloganic acid hydroxylase (7-DLH) is then used to form loganic acid by the hydroxylation of 7-deoxyloganic acid, and 41 *7-DLH* genes were identified in this step, which is 10 more genes than in the previous transcriptome data. Two methyls are added onto loganic acid to form loganin by loganic acid methyltransferase (LAMT). Eight LAMT genes were identified in the olive genome, which is similar to a previous study. Finally, secologanin is synthesized by secologanin synthase (SLS), and 39 SLS genes were obtained, which is many more than the four SLS genes in earlier transcriptome data (Fig. 7).

The hydroxytyrosol biosynthesis pathway is initiated from tyrosine and then catalyzed by polyphenol oxidase (PPO), primary amine (copper-containing) oxidase (CuAO), and tyrosine decarboxylase (TDC) to form dihydroxyphenylalanine (DOPA), *p*-hydroxyphenylacetic acid (*p*-HPAA), and tyramine, respectively. Sixteen *PPO*, eight *CuAO*, and five *TDC* genes were identified in the olive genome, with similar gene numbers to those in the previous study. DOPA is then catalyzed by DOPA decarboxylase (DDC) to produce dopamine, and five *DDC* genes were identified. Dopamine and tyramine are then oxidized by CuAO to form 4-hydroxyphenylacetic acid (4-HPA) and 3,4-dihydroxyphthalic acid (3,4-DHPA), respectively. 3,4-DHPA finally generates hydroxytyrosol through the catalysis of 10 alcohol dehydrogenases (ALDHs). In parallel, 4-HPA is catalyzed by phenylacetaldehyde reductase (PAR) to produce tyrosol. A total of nine *PAR* genes were identified in the olive genome. Finally, the formation of oleuropein from secologanin and tyrosol is catalyzed by other enzymes (Fig. 7).

The above results are based on the analysis of the whole-genome data, but some genes annotated in the genome may not be expressed in plant tissues. Combined analysis of the transcriptome and proteome of tree peony seeds on different days after pollination was conducted to better understand the transcriptional and translational regulation of seed development and oil biosynthesis, which indicated significant differences in the number and abundance of differentially expressed genes and proteins but a high level of consistency in expression patterns and metabolic pathways^18^. To study the expression levels of the above genes in different tissues of *O. europaea*, the second-generation RNA-seq transcriptome data were reanalyzed in the new genome. Samples of fruits (F), fully expanded leaves from the shoots (NL), and fully expanded leaves from the base of the stem (OL) were tested for gene expression levels (Fig. 7 and Supplementary Table S13)^19^. Fatty acid biosynthesis analysis detected 95/128 genes expressed in the tissues of F, NL, and OL (Supplementary Table S13). As in other plants, fatty acid biosynthesis in olive mainly occurs in the fruit tissue. The results of the transcriptome data also proved that the expression levels of most fatty acid synthesis-related genes in the fruits were much higher than those in the leaves. A total of 149 genes were shown to be expressed in the above tissues, accounting for three-quarters of the total number of genes (202 genes in total were identified in the oleuropein biosynthesis pathway). Heatmap analysis showed that each gene family has its own expression characteristics, implying functional differences among the family members. Using the 8HGO gene family as an example, in terms of tissue differences, the three tissues were clustered into different categories. In terms of expression level, all genes could be divided into two categories, and the expression levels of these two categories in different tissues exhibited similar expression trends.

## Discussion

Angiosperms, flowering plants, provide essential resources for human life, such as food, energy, oxygen, and materials. To date, a large number of angiosperm genomes have been sequenced^20^. Previous studies have used the shotgun sequencing method to sequence and assemble the genomes of two oil olive varieties (cultivated olive and oleaster)^5,6^. However, olive has a large genome with high heterozygosity and high repeat sequence numbers. Next-generation sequencing will thus lead to low genome quality. Previous sequencing studies obtained a contig N50 of 52 kb and scaffold N50 of 228 kb for cultivated olive and a contig N50 of 25 kb and scaffold N50 of 443 kb for oleaster (Fig. 2). Such high-quality genomes are insufficient for studying genetics and gene function in olive. To improve the quality of the olive genome, we used the Oxford Nanopore sequencing method to sequence and assemble the olive genome. A contig N50 of 4.67 Mb with a total contig length of 1.30 G and a largest contig of 25.18 Mb were obtained (Fig. 2). These obtained sequences were then assembled into 23 chromosomes by Hi-C (Fig. 3).

In addition to third-generation sequencing technology, different assembly strategies have been used to obtain high-quality genomes. The best technique for assembling genomes sequenced by Oxford Nanopore third-generation sequencing technology has not been determined^7,21^. The Canu, Wtdgb, and SMARTdenovo software packages have always been considered suitable for assembling Oxford Nanopore third-generation sequencing data^14,22^. However, it is unclear which software package is optimal for assembly. Therefore, we first used a separate assembly method to assemble the olive genome and then merged the results two by two, ultimately merging the results of the three strategies. On the basis of our assembly results, the SMARTdenovo method was optimal for use alone, while combining the results of the two methods, namely, Wtdgb+SMARTdenovo, was best. Merging of the assembly results from the three strategies produced the longest contig N50 of 4.67 M and the fewest fragments, which proved to be the best strategy for Oxford Nanopore third-generation sequencing assembly in olive (Table 1). The acquisition of this high-quality reference genome provides a good foundation for studies on the gene function and molecular breeding of olive.

Oleuropein, the most abundant olive secoiridoid, is a desirable component of high-quality olive oil and strongly influences flavor due to its bitter and pungent sensory notes^23^. Due to the particularity and importance of oleuropein, it is important to identify genes related to the oleuropein biosynthesis pathway. Thus far, the identification of oleuropein biosynthesis genes has been limited to transcriptional data, which are incomplete and not conducive to future research into the biological functions of related genes^3,24^. This study systematically identified the genes in the oleuropein biosynthesis pathway based on the high-quality oil olive genome. Compared with previous studies, the present work identified more genes that participate in the regulation of oleuropein synthesis were discovered (Fig. 6).

Very little is known about the pathway of secoiridoid synthesis at this stage, and what is known is limited to a few species, such as *Catharanthus roseus*^25^. As a consequence, the structural genes involved in this biosynthetic pathway have not been completely determined. However, the pathway from geranyl diphosphate to secologanin has been elucidated, but the subsequent reactions are unclear^26^. Based on this, we illustrated a biosynthetic pathway map containing the structural genes necessary for oleuropein synthesis (Fig. 6). A total of 202 genes were identified in the oleuropein biosynthesis pathway, which is double the number of genes identified from the previous transcriptome data. This confirmed that the obtained olive genome was nearly complete, facilitating future research into the olive genome.

The economic value, cultural value, and academic value of olive are widely acknowledged worldwide. In this study, a chromosome-level, high-quality olive genome was obtained using Oxford Nanopore third-generation sequencing and Hi-C technology, which produced large improvements over the previous version of the genome. The genome is of sufficient quality for genome-wide studies on the functions of olive genes and has provided a foundation for the molecular breeding of olive species.

## Materials and methods

### Genome survey

The physical fragmentation method (ultrasonic vibration) was used to break the extracted genomic DNA samples into fragments of ~350 bp, from which three small-fragment sequencing libraries were constructed through the steps of end repair, addition of A, addition of adapters, target fragment selection, and PCR. The libraries were then sequenced using the NovaSeq 6000 system. To determine whether the extracted sample DNA was contaminated, 10,000 single-end reads were randomly selected from the three 350 bp libraries obtained by sequencing and BLAST-compared with the Nt database^12^. The three 350-bp libraries obtained by Illumina sequencing were compared with the chloroplast sequences (NC_015623.1, 155,896 bp) of oleaster, a relative of olive, to determine whether there was nonnuclear DNA contamination^13^. The library data were used to construct a *k-mer* distribution map with *k* = 21 and to assess the genome size, ratio of repeated sequences, and heterozygosity. The *k-mer* analysis was carried out using “*k-mer* freq stat” software (developed by Biomarker 120 Technologies Corporation, Beijing, China). Genome size (G) was estimated based on the following formula: G = *k-mer* number/average *k-mer* depth, where *k-mer* number = total *k-mers*-abnormal k-mers (with too low or too high frequency).

### Genome sequencing and de novo assembly

Leaf samples of *O. europaea* cv. ‘*Arbequina*’ were collected in the olive grove of the Research Institute of Forestry, Chinese Academy of Forestry, in Mianning, Sichuan Province. The genome was sequenced using the Oxford Nanopore third-generation sequencing platform. Clean data were corrected by Canu software, following which Wtdbg (https://github.com/ruanjue/wtdbg), SMARTdenovo (https://github.com/ruanjue/smartdenovo)^14,27,28^, and Canu were used for genome assembly based on the corrected data, and then the genomes assembled by the three software packages were merged by Quickmerge software (https://github.com/mahulchak/quickmerge). Racon software was used to perform three rounds of correction on the integrated genome, and then the next-generation DNA-seq data were used to perform three rounds of correction using Pilon software, ultimately obtaining the genome sequence^29^. CEGMA and BUSCO were used to assess the completeness of the genome assembly^15,30^.

The repeat sequence database of the olive genome was constructed using two software programs, namely, LTR_FINDER and RepeatScout^31,32^. PASTEClassifier was used to categorize the database, which was then combined with the Repbase database as the final repeat sequence database^33,34^. RepeatMasker was used to predict the repeat sequence of the olive genome based on the constructed repeat sequence database^35^. Genscan, Augustus, GlimmerHMM, GeneID, and SNAP software were used to make a de novo prediction of the genetic structure of the genome^36–39^; GeMoMa was used to make predictions based on homologous species^40^; and then EVM software was used to integrate the prediction results^41^. Hisat and Stringtie software^42,43^ were used for transcript assembly (accession numbers: SRR10743047, SRR10743049, SRR10743048, SRR10743044, SRR10743045, SRR10743046, SRR10743041, SRR10743042, and SRR10743043), TransDecoder (http://transdecoder.github.io) and GeneMarkS-T software were used for gene prediction^44^.

### Hi-C library construction and chromosome assembly

The type of Hi-C library construction and sequencing was in situ Hi-C, which mainly includes cell crosslinking, endonuclease digestion, biotinylation, cyclization, DNA purification, capture, and sequencing^45,46^. Fresh tissues (leaves) were crossed-linked with formaldehyde, and cross-linked DNA was then digested by Hind III restriction enzyme. The sticky ends of these fragments were end-repaired, marked with biotin, and then blunt-end proximity-ligated to generate circular molecules. Subsequently, these circular DNA molecules were fragmented into 300–500 bp fragments, and DNA ends were sheared, enriched by biotin pulldown and processed for paired-end sequencing (150-bp paired-end). After library construction had been completed, the library concentration and insert size were detected using a Qubit 2.0 fluorimeter and Agilent 2100 bioanalyzer, respectively, and the effective concentration of the library was accurately quantified using quantitative PCR to ensure library quality. The Illumina NovaSeq 6000 platform was then used for high-throughput sequencing with a read length of PE150. The obtained Hi-C data were used for chromosome-level assemblies. The draft contigs were divided into fragments with a length of 50 kb and clustered by LACHESIS software using valid interaction read pairs^16^. We assessed the quality of each fragment with HiCPro (v2.8.1)^35^ and removed duplicates^47^, and Hi-C data were then mapped to the segments using BWA (v0.7.10-r789) software^48^. The uniquely mapped data were retained for scaffold assembly using LACHESIS software with parameters CLUSTER_N = 10, CLUSTER_MIN_RE_SITES = 48, ORDER_MIN_N_RES_IN_TRUN = 14, CLUSTER_MAX_LINK_DENSITY = 2, CLUSTER_NONINFORMATIVE_RATIO = 2, and ORDER_MIN_RES_IN_SHREDS = 15.

### Gene cluster analysis and phylogenetic tree construction

Orthofinder software was used to classify the protein sequences of 12 species into families (the alignment method used was diamond, and the alignment e-value was 0.001), and the PANTHER database was used to annotate the obtained gene families^49,50^. Finally, GO and KEGG enrichment analyses were performed for the olive-specific gene families^51^. MAFFT was used to compare each single-copy gene family sequence (parameter: localpair -maxiterate 1000), and then Gblocks (parameter: b5 = h) was used to remove regions with poor sequence alignment or large differences. All the gene family sequences were connected end-to-end to obtain a supergene^52,53^. IQFinder’s built-in model detection tool ModelFinder was used for model detection, and the best model obtained was JTT + F + I + G4. This best model was then used to construct an evolutionary tree using the maximum likelihood (ML) method, with the number of bootstrap replicates set to 1,000^54^. MCMCTREE, a software package that comes with PAML, was used to calculate divergence times^55^.

### Gene family expansion and contraction analysis

CAFE (Computational Analysis of gene Family Evolution) software was used to analyze divergence times and gene family expansion and contraction^56^. The results of evolutionary tree and gene family clustering were used to estimate the number of gene families of the ancestors in each phylogenetic tree branch, thereby predicting gene family contraction and expansion. The criterion for defining significant expansion or contraction was a *P*-value < 0.05.

### Positive selection analysis

The CodeML module in PAML was used for positive selection analysis^55^. Single-copy genes of *C. sinensis*, *H. annuus*, *O. europaea*, *O*. *europaea*, *L. sylvestris*, and *S. indicum* were obtained, and the protein sequence of each gene family was compared using MAFFT (parameter: localpair -maxiterate 1000). The “chi2” program in the PAML program was used to perform likelihood ratio tests on Model A (assuming that the foreground branch *ω* was in a positive choice, i.e., *ω* > 1) and the null model (meaning that the *ω* value of any site was not allowed to be >1), with significance assessed at *P* < 0.01. The Bayesian method (BEB, Bayes empirical Bayes method) was used to obtain positive selection sites (greater than 0.95 is usually considered significantly positively selected sites), and the genes receiving significant positive selection were ultimately obtained.

### Synteny analysis

Diamond software was used to compare the gene sequences of the two species to determine similar gene pairs (e < 1e − 5, C score > 0.5, where JCVI software was used to filter the C score value)^57^. Next, MCScanX software was used to determine whether similar gene pairs were adjacent on the chromosome, ultimately obtaining all the genes in the synteny block^58^. Samples for RNA-seq discussed in the “Identification of oleuropein and fatty acid biosynthesis genes in olive” section were analyzed according to a previous study^19^.

## Supplementary information

supplementary figures and tables

## Data Availability

The whole-genome sequence data reported in this paper have been deposited in the Genome Warehouse in the National Genomics Data Center (NGDC), Beijing Institute of Genomics (China National Center for Bioinformation), and Chinese Academy of Sciences^59^ under the BioProject accession number PRJCA003222 and the Biosample number SAMC206766. Clean ONT sequence data were deposited in the Genome Sequence Archive (GSA) of NGDC under the accession number CRA003087.
