# Supplementary material for: De novo assembly of a new Olea europaea genome accession using nanopore sequencing
Source: Hortic Res. 2021 Apr 1;8:64. doi: 10.1038/s41438-021-00498-y (PMC8012569; doi:10.1038/s41438-021-00498-y)
Supplement: Supplementary file 1 — supplementary figures and tables [file 41438_2021_498_MOESM1_ESM.docx]

Supplementary Figures


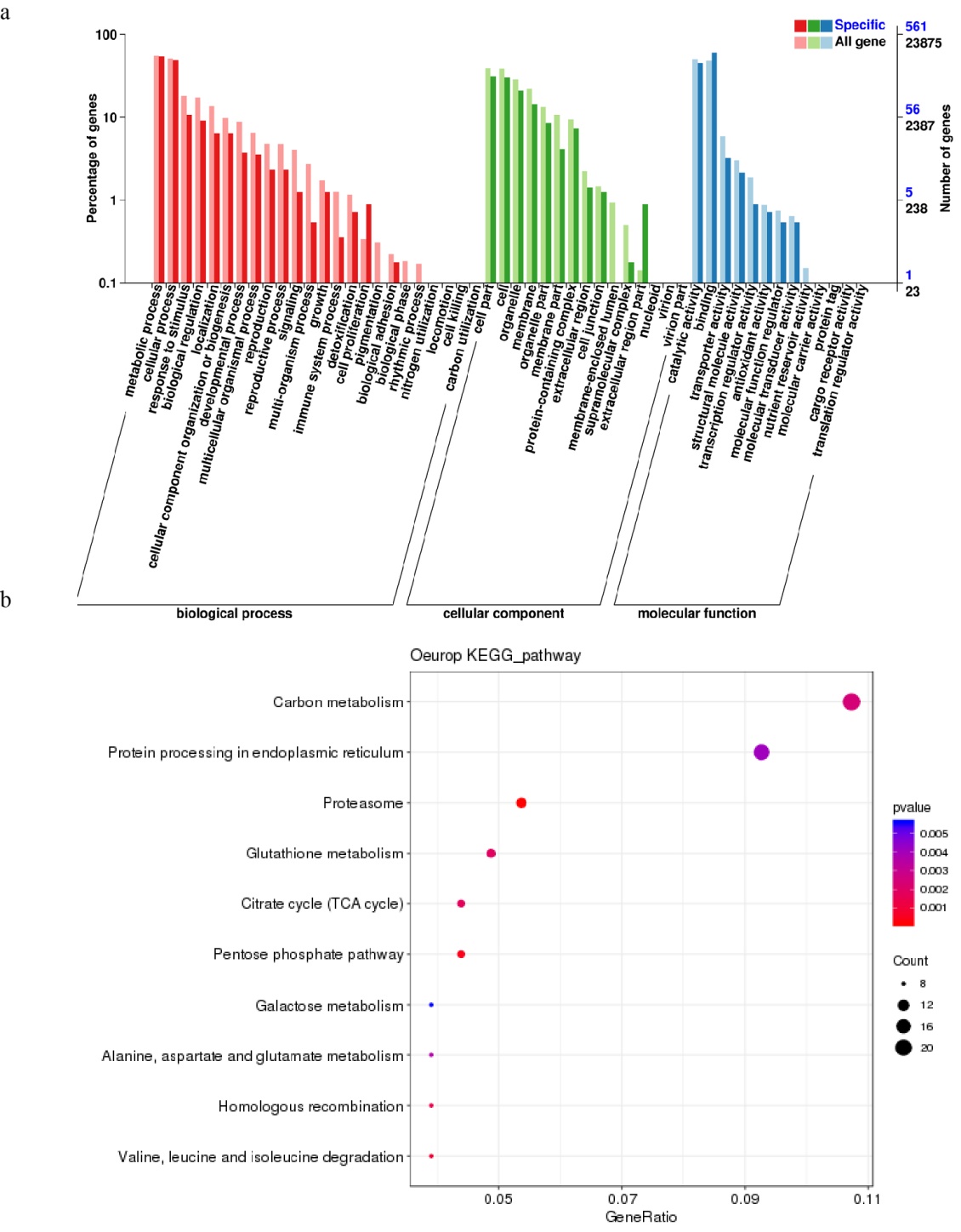


**Fig. S1 GO annotations and KEGG pathway analysis of** **specific gene families in *O. europaea*. a** GO annotations analysis of specific gene families in *O. europaea*. **b.** KEGG pathway analysis of specific gene families in *O. europaea.*


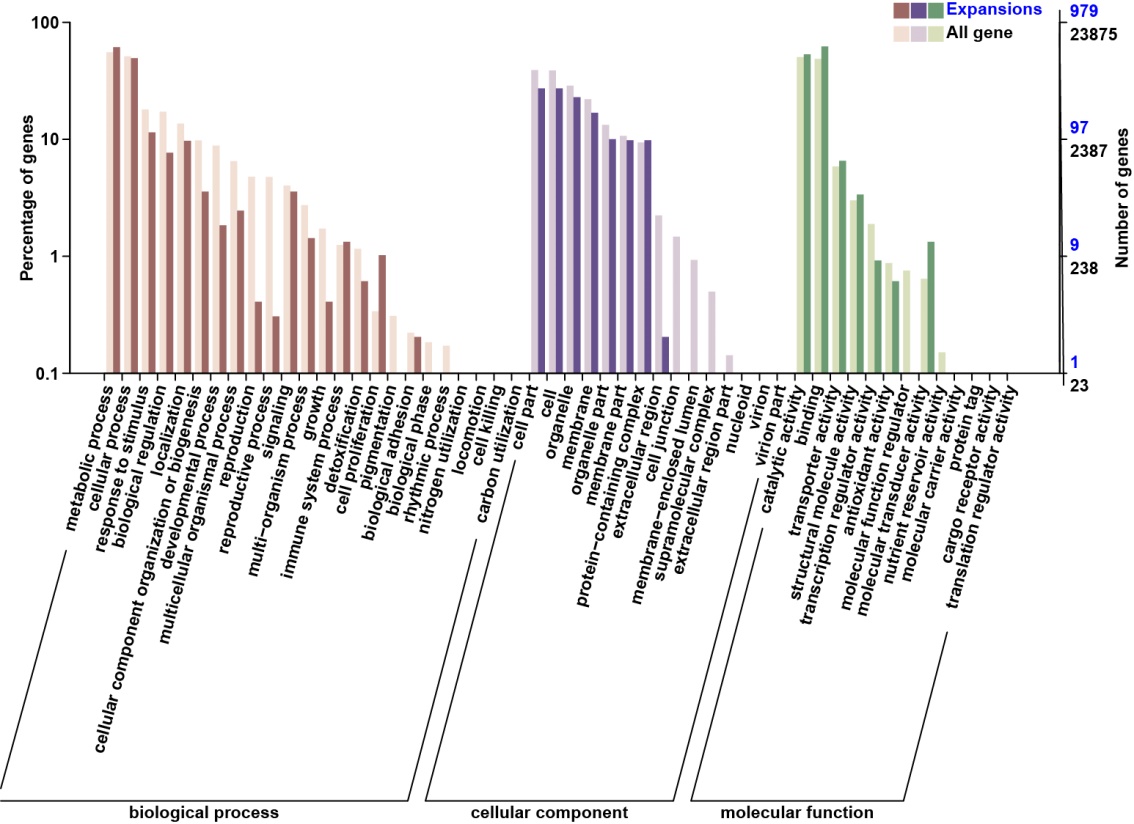


**Fig. S2 GO annotations of** **expanded genes in *O. europaea* .**

**
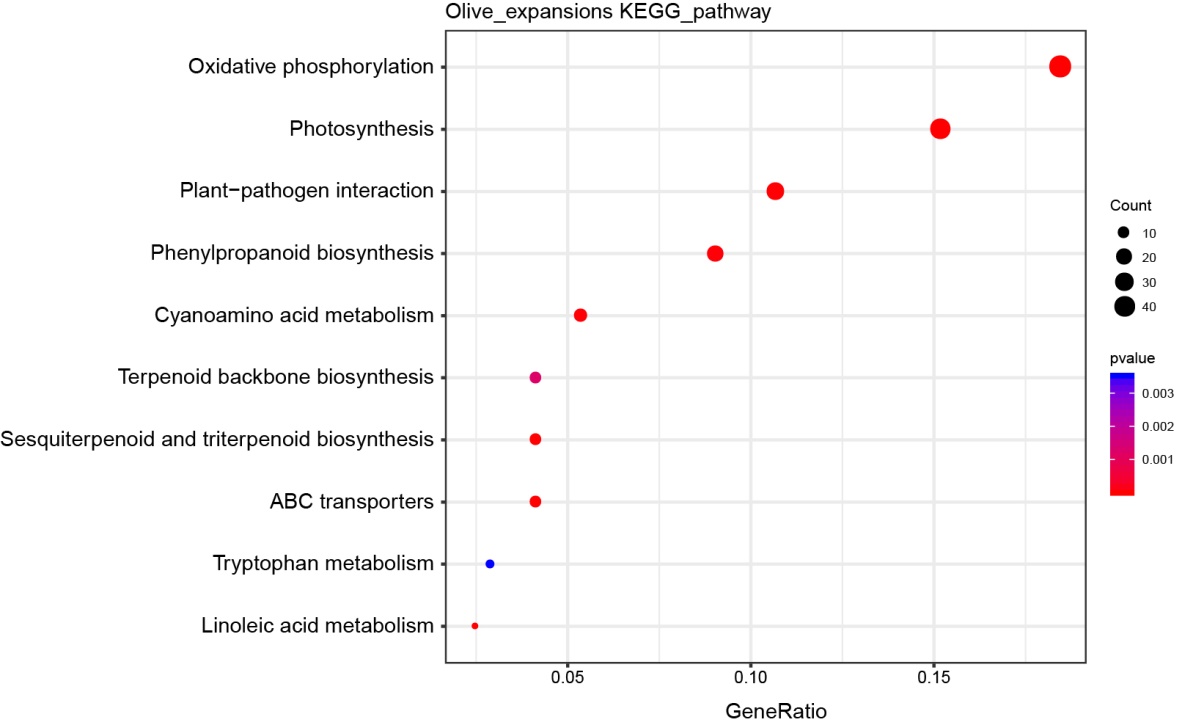
**

**Fig. S3 The KEGG pathway analysis of** **expanded genes in *O. europaea* .**

Supplementary Tables

**Table S1 Illumina sequencing data statistics**. The Illumina RNA-seq platform was then used for high-throughput sequencing with a read length of PE150. Library: the sequencing library of surveyData (Gb): the amount of sequencing data of the corresponding sequencing library; Depth (×): the depth of sequencing; Q20 (%): the proportion of bases with a sequencing quality value of 20 or more; Q30 (%): The ratio of bases whose sequencing quality value is above 30.

| Library | Data (Gb) | Depth (×) | Q20 (%) | Q30 (%) |
| --- | --- | --- | --- | --- |
| 350 bp_1 | 31.09 | 24.37 | 96.35 | 91.22 |
| 350 bp_2 | 33.26 | 26.07 | 96.34 | 91.1 |
| 350 bp_3 | 32.12 | 25.18 | 96.4 | 91.21 |
| Total | 96.48 | 75.61 | --- | --- |

**Table S2 ONT sequencing data statistics.** SeqNum: total read number of sequencing data; SumBase(bp): total base number of sequencing data; N50Len: N50 length of sequencing data reads; N90Len: N90 length of sequencing data reads; MeanLen: the average length of sequencing data reads; MaxLen: the longest length of sequencing data reads; MeanQual: average quality value.

| Data type | SeqNum | SumBase | N50Len | N90Len | MeanLen | MaxLen | MeanQual |
| --- | --- | --- | --- | --- | --- | --- | --- |
| Raw data | 9,009,932 | 146,825,799,392 | 30,203 | 17,916 | 16,295 | 1,090,232 | 5.52 |
| Clean data | 4,708,203 | 128,588,756,353 | 30,890 | 19,846 | 27,311 | 962,647 | 8.2 |

**Table S3 Length distribution of clean reads.** Length: refers to the various length ranges of reads; ReadsNum: the number of sequences; TotalLength: the total length of the sequence; Percent: the ratio of the number of sequences to the total number; AveLength: the average length of the sequence.

| Length | Reads Number | Total Length (bp) | Percent | Average Length (bp) |
| --- | --- | --- | --- | --- |
| 2,000~5,000 | 213,445 | 726,347,290 | 0.56% | 3,402.97 |
| 5,000~10,000 | 279,987 | 2,099,164,360 | 1.63% | 7,497.36 |
| 10,000~20,000 | 670,481 | 10,376,641,224 | 8.06% | 15,476.41 |
| 20,000~30,000 | 1,875,179 | 47,145,504,879 | 36.66% | 25,141.86 |
| 30,000~40,000 | 949,228 | 32,462,521,936 | 25.24% | 34,198.86 |
| 40,000~50,000 | 439,023 | 19,530,237,597 | 15.18% | 44,485.68 |
| 50,000~60,000 | 202,342 | 10,962,129,640 | 8.52% | 54,176.24 |
| 60,000~70,000 | 60,029 | 3,832,712,861 | 2.98% | 63,847.68 |
| 70,000~80,000 | 13,384 | 986,975,104 | 0.76% | 73,742.90 |
| ≥80,000 | 5,105 | 466,521,462 | 0.36% | 91,385.20 |

**Table S4 Data contamination assessment of three 350 bp libraries**

|  | Species | Aligned Percentage(%) |
| --- | --- | --- |
| 350 bp_1 | *Olea europaea* | 66.19 |
|  | *Hesperelaea palmeri* | 8.15 |
|  | *Vitis vinifera* | 1.37 |
|  | *Artemisia fukudo* | 1.2 |
|  | *Sesamum indicum* | 1.16 |
|  | *Osmanthus fragrans* | 1.12 |
|  | Others | 20.24 |
| 350 bp_2 | *Olea europaea* | 64.99 |
|  | *Hesperelaea palmeri* | 8.15 |
|  | *Sesamum indicum* | 1.69 |
|  | *Vitis vinifera* | 1.57 |
|  | *Scrophularia dentata* | 1.27 |
|  | *Osmanthus fragrans* | 1.06 |
|  | Others | 20.52 |
| 350 bp_3 | *Olea europaea* | 70.96 |
|  | *Hesperelaea palmeri* | 5.75 |
|  | *Osmanthus fragrans* | 1.67 |
|  | *Sesamum indicum* | 1.4 |
|  | *Vitis vinifera* | 1.35 |
|  | Other | 18.24 |

**Table S5 SOAP alignment with the three 350 bp libraries from Illumina sequencing of the chloroplast sequences of olive (the oleaster sequence).**

|  | Type | Reads aligned | Total reads | Percentage (%) |
| --- | --- | --- | --- | --- |
| 350 bp_1 | Paired-read | 4,934,888 | 415,213,924 | 2.38 |
|  | Single-read | 2,431,268 | 415,213,924 | 1.17 |
| 350 bp_2 | Paired-read | 5,385,938 | 444,101,048 | 2.43 |
|  | Single-read | 2,450,201 | 444,101,048 | 1.1 |
| 350 bp_3 | Paired-read | 2,785,904 | 428,954,336 | 1.3 |
|  | Single-read | 1,519,812 | 428,954,336 | 0.71 |

**Table S6 BUSCO evaluation of assembled genome.** Species: species information; Complete BUSCOs: the number of complete genes; Complete and single-copy BUSCOs: the number of single-copy genes; Complete and duplicated BUSCOs: the number of multiple copy genes; Fragmented BUSCOs: predicted number of incomplete genes; Missing BUSCOs: not predicted number of genes

| Cultivar |  | Complete BUSCOs(C) | Complete and single-copy BUSCOs(S) | Complete and duplicated BUSCOs(D) | Fragmented BUSCOs(F) | Missing BUSCOs(M) | Total Lineage BUSCOs |
| --- | --- | --- | --- | --- | --- | --- | --- |
| *Arbequina* | Genome | 1521 (94.24%) | 1192 (73.85%) | 329 (20.38%) | 39 (2.42%) | 54 (3.35%) | 1614 |
|  | gene sets | 1499 (92.87%) | 1291 (79.99%) | 208 (12.89%) | 35 (2.17%) | 80 (4.96%) | 1614 |
| *Farga* | Genome | 1501 (92.99%) | 1269 (78.62%) | 293 (18.15%) | 25 (1.55%) | 27 (1.67%) | 1614 |
|  | gene sets | 1510 (93.55%) | 771 (47.77%) | 796 (49.32%) | 19 (1.18%) | 28 (1.73%) | 1614 |
| *sylvestris* | Genome | 1380 (85.50%) | 767 (47.52%) | 613 (37.98%) | 108 (6.69%) | 126 (7.81%) | 1614 |
|  | gene sets | 1376 (85.25%) | 1224 (75.84%) | 152 (9.42%) | 90 (5.58%) | 148 (9.17%) | 1614 |

**Table S7 CEGMA evaluation of assembled genome.** Species: species information; Number of 458 CEGs* present in assembly: the number of genes found in the assembled genome; % of 458 CEGs present in assemblies: the proportion of conserved genes; Number of 248 highly conserved CEGs present: the number of highly conserved genes;% of 248 highly conserved CEGs present: The proportion of highly conserved genes.

| Number of 458 CEG* present in assembly | % of 458 CEGs present in assemblies | Number of 248 highly conserved CEGs present | % of 248 highly conserved CEGs present |
| --- | --- | --- | --- |
| 438 | 95.63% | 225 | 90.73% |

**Table S8 Genomic statistics after assembly of Hi-C.**

| Scaffold number | Scaffold length (bp) | Scaffold N50 (bp) | Scaffold N90 (bp) | Scaffold max (bp) | Gap total length (bp) |
| --- | --- | --- | --- | --- | --- |
| 962 | 1,102,969,454 | 42,601,851 | 279,924 | 68,066,766 | 138,400 |

**Table S9 Hi-C assembly statistics.**

| Group | Cluster Num | Cluster Len(bp) | Order Num | Order Len(bp) |
| --- | --- | --- | --- | --- |
| Chr01 | 99 | 41,600,470 | 80 | 40,020,404 |
| Chr02 | 101 | 47,730,346 | 71 | 45,188,063 |
| Chr03 | 54 | 40,634,898 | 24 | 37,512,869 |
| Chr04 | 65 | 38,980,629 | 54 | 38,198,886 |
| Chr05 | 47 | 29,904,147 | 40 | 29,304,931 |
| Chr06 | 106 | 56,799,367 | 82 | 54,943,300 |
| Chr07 | 74 | 44,846,003 | 62 | 42,595,751 |
| Chr08 | 66 | 35,781,243 | 40 | 32,675,436 |
| Chr09 | 79 | 37,359,505 | 63 | 36,016,850 |
| Chr10 | 171 | 76,617,400 | 104 | 68,056,466 |
| Chr11 | 109 | 61,239,810 | 63 | 57,218,697 |
| Chr12 | 118 | 52,405,078 | 93 | 50,506,245 |
| Chr13 | 90 | 54,003,902 | 73 | 52,332,911 |
| Chr14 | 78 | 37,796,640 | 59 | 35,389,338 |
| Chr15 | 72 | 46,312,966 | 57 | 43,964,291 |
| Chr16 | 94 | 38,762,891 | 70 | 36,321,892 |
| Chr17 | 63 | 45,065,125 | 46 | 43,296,874 |
| Chr18 | 102 | 48,527,813 | 89 | 47,171,929 |
| Chr19 | 96 | 53,753,178 | 60 | 50,601,053 |
| Chr20 | 73 | 32,640,312 | 62 | 31,763,455 |
| Chr21 | 51 | 28,668,958 | 37 | 27,602,399 |
| Chr22 | 46 | 37,330,271 | 31 | 36,198,904 |
| Chr23 | 60 | 40,838,850 | 47 | 39,631,878 |
| Total(Ratio %) | 1914(81.59) | 1027599802(93.18) | 1407(73.51) | 976512822(95.03) |

**Table S10 Satatistic of repeate sequences.** Type: type of repetitive sequence; Number: number of repetitive sequence; Length: total length of repetitive sequence; Rate(%): the proportion of repetitive sequence in the total genome.

| Type | Number | Length | Rate(%) |
| --- | --- | --- | --- |
| ClassI | 1,230,412 | 627,330,829 | 56.88 |
| ClassI/DIRS | 61,730 | 46,116,651 | 4.18 |
| ClassI/LARD | 414,019 | 142,735,185 | 12.94 |
| ClassI/LINE | 30,745 | 10,233,803 | 0.93 |
| ClassI/LTR/Copia | 389,564 | 222,801,577 | 20.2 |
| ClassI/LTR/Gypsy | 292,928 | 226,571,566 | 20.54 |
| ClassI/LTR/Unknown | 14,566 | 15,099,957 | 1.37 |
| ClassI/PLE | 969 | 648,987 | 0.06 |
| ClassI/SINE | 8,660 | 1,643,556 | 0.15 |
| ClassI/TRIM | 15,267 | 18,477,605 | 1.68 |
| ClassI/Unknown | 1,964 | 1,310,897 | 0.12 |
| ClassII | 141,562 | 58,311,236 | 5.29 |
| ClassII/Crypton | 12 | 616 | 0 |
| ClassII/Helitron | 9,634 | 5,047,934 | 0.46 |
| ClassII/MITE | 6,200 | 1,273,831 | 0.12 |
| ClassII/Maverick | 499 | 342,382 | 0.03 |
| ClassII/TIR | 117,810 | 47,857,812 | 4.34 |
| ClassII/Unknown | 7,407 | 4,342,825 | 0.39 |
| PotentialHostGene | 40,007 | 11,149,520 | 1.01 |
| SSR | 6,817 | 5,850,832 | 0.53 |
| Unknown | 396,787 | 99,378,618 | 9.01 |
| Total | 1,815,585 | 743,103,344 | 67.37 |

**Table S11 Genome annotation statistics**

| Database | Annotated number | Percentage (%) |
| --- | --- | --- |
| GO | 23,875 | 44.61 |
| KEGG | 16,298 | 30.45 |
| KOG | 29,171 | 54.51 |
| TrEMBL | 50,837 | 94.99 |
| Nr | 50,898 | 95.1 |
| All_Annotated | 50,969 | 95.24 |

**Table 12 Genes classification statistics.**

| Name | Number of genes | Number of genes in orthogroups | Number of unassigned genes | Number of species-specific orthogroups | Number of genes in species-specific orthogroups |
| --- | --- | --- | --- | --- | --- |
| *A. hypogaea* | 36,450 | 28,793 | 7,657 | 1,522 | 7,314 |
| *A. thaliana* | 27,379 | 18,520 | 8,859 | 2,389 | 6,452 |
| *A. trichopoda* | 26,846 | 18,688 | 8,158 | 1,107 | 4,456 |
| *C. sinensis* | 36,951 | 25,350 | 11,601 | 998 | 3,007 |
| *G. max* | 56,044 | 48,453 | 7,591 | 1,253 | 4,912 |
| *H. annuus* | 52,243 | 39,347 | 12,896 | 3,652 | 13,145 |
| *O. europaea cv ‘arbequina’* | 53,518 | 48,834 | 4,684 | 806 | 2,084 |
| *O. europaea var. sylvestris* | 39,875 | 37,197 | 2,678 | 159 | 387 |
| *O. sativa* | 42,189 | 24,348 | 17,841 | 4,170 | 13,291 |
| *P. trichocarpa* | 41,335 | 34,803 | 6,532 | 841 | 3,037 |
| *R. communis* | 31,220 | 20,786 | 10,434 | 619 | 1,737 |
| *S. indicum* | 27148 | 23842 | 3306 | 533 | 3054 |

**Table S13 Expression level of fatty acids biosynthesis genes in olive.** BC, biotin carboxylase; BCCP, carboxylated biotin carrier protein; CT, carboxyltransferase; MAT, malonyl-CoA ACP transacylase; KAS, ketoacyl-ACP synthase; FATA/B, acyl-ACP thioesterase A/B; KAR, ketoacyl-ACP reductase; HAD, hydroxyacyl-ACP dehydrase; EAR, enoyl-ACP reductase; LACS, long-chain acyl-CoA synthetase; KCS, Ketoacyl-CoA synthase;

|  |  | Fruit | New leave | Old leave |
| --- | --- | --- | --- | --- |
| BC | EVM0017802 | 7.53 | 12.14 | 15.01 |
|  | EVM0012415 | 698.91 | 26.09 | 15.30 |
|  | EVM0008752 | 6.63 | 6.33 | 9.85 |
|  | EVM0051454 | 60.54 | 36.90 | 18.78 |
|  | EVM0049907 | 671.91 | 31.80 | 14.51 |
| BCCP | EVM0028970 | 145.91 | 3.43 | 0.03 |
|  | EVM0009821 | 0.98 | 0.17 | 0.66 |
|  | EVM0000308 | 30.28 | 10.02 | 7.63 |
|  | EVM0053294 | 447.42 | 45.46 | 0.23 |
|  | EVM0055867 | 1207.11 | 21.67 | 0.55 |
|  | EVM0017596 | 19.79 | 14.64 | 16.69 |
|  | EVM0052040 | 2.21 | 1.14 | 2.42 |
|  | EVM0051166 | 1.79 | 1.43 | 2.12 |
|  | EVM0039492 | 143.82 | 11.59 | 0.14 |
|  | EVM0044315 | 21.13 | 10.09 | 10.58 |
|  | EVM0024205 | 22.10 | 11.81 | 4.59 |
|  | EVM0021024 | 14.19 | 8.59 | 2.10 |
|  | EVM0044338 | 61.43 | 4.79 | 1.24 |
| CT | EVM0024686 | 5.24 | 6.51 | 5.29 |
| MAT | EVM0050869 | 19.09 | 2.86 | 0.40 |
|  | EVM0047737 | 483.64 | 31.13 | 15.76 |
|  | EVM0020489 | 4.32 | 0.50 | 0.09 |
| KAS | EVM0042037 | 85.24 | 6.11 | 3.69 |
|  | EVM0015797 | 123.33 | 14.64 | 15.87 |
|  | EVM0024043 | 58.89 | 28.35 | 41.26 |
|  | EVM0043457 | 52.28 | 16.64 | 10.52 |
|  | EVM0039115 | 21.67 | 27.98 | 13.16 |
| FATA | EVM0031236 | 120.64 | 22.21 | 2.58 |
| FATB | EVM0009935 | 19.95 | 46.72 | 16.05 |
|  | EVM0024086 | 46.25 | 133.97 | 25.34 |
| KAR | EVM0034348 | 5.11 | 25.90 | 19.11 |
|  | EVM0027324 | 5.58 | 5.64 | 7.13 |
|  | EVM0047543 | 1224.98 | 63.51 | 0.54 |
|  | EVM0039457 | 3.76 | 0.00 | 0.00 |
|  | EVM0032567 | 50.45 | 0.41 | 0.18 |
|  | EVM0037328 | 208.66 | 41.12 | 24.73 |
|  | EVM0036541 | 13.40 | 9.86 | 21.09 |
|  | EVM0021695 | 0.07 | 10.42 | 0.04 |
|  | EVM0031337 | 69.80 | 13.12 | 7.77 |
|  | EVM0030047 | 429.26 | 0.50 | 0.18 |
|  | EVM0049914 | 15.23 | 1.58 | 0.24 |
|  | EVM0050459 | 108.31 | 32.06 | 40.73 |
|  | EVM0047991 | 12.89 | 12.08 | 6.56 |
|  | EVM0012922 | 571.56 | 10.00 | 18.90 |
|  | EVM0055613 | 7.00 | 19.08 | 12.37 |
|  | EVM0017814 | 1266.06 | 30.98 | 54.77 |
|  | EVM0036349 | 2722.31 | 30.27 | 50.19 |
|  | EVM0029586 | 14.57 | 0.77 | 0.23 |
| HAD | EVM0039430 | 227.79 | 11.85 | 5.15 |
|  | EVM0003519 | 390.07 | 27.82 | 19.42 |
| EAR | EVM0026081 | 192.04 | 14.24 | 0.09 |
|  | EVM0011889 | 10.14 | 26.72 | 33.21 |
|  | EVM0058753 | 325.07 | 32.18 | 4.70 |
| LACS | EVM0045919 | 3.28 | 0.30 | 0.00 |
|  | EVM0034755 | 70.71 | 0.64 | 0.02 |
|  | EVM0048591 | 11.68 | 37.60 | 67.54 |
|  | EVM0056318 | 0.58 | 3.52 | 0.61 |
|  | EVM0021878 | 0.18 | 34.62 | 4.10 |
|  | EVM0047780 | 32.78 | 17.70 | 33.90 |
|  | EVM0061605 | 70.46 | 25.02 | 36.46 |
|  | EVM0051417 | 35.16 | 42.36 | 57.41 |
|  | EVM0025938 | 20.14 | 56.63 | 60.07 |
|  | EVM0018253 | 68.77 | 300.89 | 9.36 |
| KCS | EVM0003288 | 2.54 | 0.66 | 0.39 |
|  | EVM0020395 | 0.00 | 0.78 | 0.84 |
|  | EVM0059698 | 7.73 | 292.82 | 14.93 |
|  | EVM0021728 | 0.17 | 2.61 | 0.68 |
|  | EVM0008899 | 0.01 | 48.65 | 0.06 |
|  | EVM0007338 | 15.78 | 5.92 | 6.42 |
|  | EVM0018853 | 5.24 | 12.17 | 0.19 |
|  | EVM0023101 | 0.00 | 8.04 | 0.58 |
|  | EVM0026451 | 0.00 | 2.73 | 2.95 |
|  | EVM0059848 | 0.00 | 108.19 | 0.20 |
|  | EVM0042419 | 0.00 | 4.59 | 3.61 |
|  | EVM0051678 | 3.81 | 5.89 | 9.13 |
|  | EVM0027995 | 20.57 | 119.86 | 10.49 |
|  | EVM0013706 | 1.22 | 1.73 | 0.30 |
|  | EVM0003502 | 3.61 | 8.00 | 10.55 |
|  | EVM0014055 | 7.01 | 6.57 | 5.53 |
|  | EVM0003783 | 1.91 | 7.21 | 3.49 |
|  | EVM0015983 | 0.00 | 113.63 | 0.56 |
|  | EVM0044700 | 4.30 | 13.53 | 6.94 |
|  | EVM0061058 | 28.21 | 174.47 | 8.23 |
|  | EVM0017753 | 3.46 | 25.42 | 4.20 |
|  | EVM0039273 | 3.44 | 1.46 | 0.21 |
|  | EVM0034488 | 13.88 | 24.17 | 7.06 |
|  | EVM0038805 | 1.37 | 7.75 | 0.43 |
